# Psychosocial outcomes after varying risk management strategies in women at increased familial breast cancer risk: a mixed methods study of patient and partner outcomes

**DOI:** 10.1308/rcsann.2023.0042

**Published:** 2023-07-17

**Authors:** J Morgan, E MacInnes, S Erskine, SJ Walters, J Cook, K Collins, L Wyld

**Affiliations:** ^1^ University of Sheffield, UK; ^2^ Leeds Teaching Hospitals NHS Foundation Trust, UK; ^3^ East of England School of General Practice, Norwich, UK; ^4^ University of Sheffield, UK; ^5^ Sheffield Childrens NHS Foundation Trust, UK; ^6^ Sheffield Hallam University, UK

**Keywords:** *BRCA* gene carriers, Risk-reducing surgery, Surveillance, Screening, Decision making, Psychosocial outcomes

## Abstract

**Introduction:**

Female carriers of *BRCA1/2* genes have an increased lifetime risk of breast cancer. Options for managing risk include imaging surveillance or risk-reducing surgery (RRS). This mixed methods study aimed to identify factors affecting risk-management decisions and the psychosocial outcomes of these decisions for high-risk women and their partners.

**Methods:**

Semi-structured qualitative interviews were performed with women at high breast cancer risk who had faced these choices. Partners were also interviewed. Analysis used a framework approach. A bespoke questionnaire was developed to quantify and explore associations.

**Results:**

A total of 32 women were interviewed. Of these, 27 had partners of whom 7 (26%) agreed to be interviewed. Four main themes arose: perception of risk and impact of increased risk; risk-management strategy decision-making; impact of risk-management strategy; support needs and partner relationship issues. The questionnaire response rate was 36/157 (23%). Decision satisfaction was high in both surveillance and RRS groups. Relationship changes were common but not universal. Common causes of distress following RRS included adverse body image changes. Both groups experienced generalised and cancer-specific anxiety. Drivers for surgery included having children, deaths of close family from breast cancer and higher levels of cancer anxiety.

**Conclusions:**

Levels of psychosocial and decision satisfaction were high for women choosing both RRS and surveillance but, for a minority, risk-reducing measures result in long-term psychosocial morbidity. Efforts to recognise women at increased risk of psychological morbidity may allow targeted support.

## Introduction

Pathogenic variants of the *BRCA1* and *BRCA2* genes confer a 72% and 69% risk of a woman developing breast cancer by the age of 80 years, respectively, with the average age at diagnosis some 20 years younger than for sporadic cancers.^1^ For *BRCA1* carriers there is also an increased risk of developing a more aggressive cancer phenotype (triple negative) with a worse prognosis.^2,3^

Increased awareness of familial risk and improved availability of testing mean that more women are being identified as “at risk” when they have not had cancer themselves. However, becoming risk aware may have profound psychological consequences.^4,5^

Women identified as being at high risk of developing breast cancer may be offered a number of risk-management strategies, broadly divided into surveillance (plus or minus chemoprevention) or risk-reducing surgery (RRS).

Due to the relative insensitivity of mammography in younger women,^6^ magnetic resonance imaging (MRI) surveillance is often used and is effective in the early diagnosis of cancer with improved cancer detection compared with mammography alone.^7,8^ However, MRI surveillance is associated with a high recall rate for nonsignificant abnormalities,^9^ and even if a cancer is diagnosed at an earlier stage when it is more likely to be cured, it is associated with cancer-related anxiety and substantial treatment-related morbidity (which may include mastectomy, radiotherapy, chemotherapy and consequent fertility issues). Anxiety symptoms are reported to increase around the time of scans and results.^10^ Whereas surveillance does not prevent the development of cancer, a fact that causes some women considerable anxiety, this strategy is associated with a survival benefit and is likely to reduce the burden of treatment due to diagnosis at an earlier stage.^11^

Risk-reducing mastectomy (RRM) offers the most effective risk reduction for these women, estimated at between 85% and 100%.^12–14^ However, this is major surgery that may have variable cosmetic outcomes and a risk of potentially severe physical and psychological complications.^12–16^ Most women undergo RRM without developing major emotional distress, but postoperative distress scores are frequently raised to clinically significant levels.^17^ Up to half of women undergoing RRS will suffer negative effects on body-image and sexuality,^18^ although this finding is variable and satisfaction is generally been reported to be high.^19^

Risk-reducing oophorectomy, if undertaken in a premenopausal woman, may also result in a reduction in breast cancer risk for women who have completed their families if *BRCA2* gene carriers.^20^ Use of tamoxifen and aromatase inhibitors may also be added to the risk-management strategy, although once again is more likely to be effective in *BRCA2* gene carriers due to the biological subtypes of cancer predisposed to.

The impact on partners of affected women has rarely been explored. A systematic review of men’s experiences of their partners’ mastectomy found that men struggled to talk openly to their partners about body image after surgery, with lack of communication leading to conflict and poor psychological wellbeing.^21^ Another study using an online survey attached to cancer support boards found that partners reported changes in intimacy, attraction and communication after disclosure of familial breast cancer risk. Concern about postsurgical appearance, attraction, health and concern about sexual relationship were noted in men whose partners were awaiting surgery.^22^

This mixed methods study aimed to identify the psychosocial outcomes for women at high risk of breast cancer and their partners, and assess factors impacting on risk-management decisions, and decision satisfaction.

## Methods

### Study design

The study followed an exploratory, sequential, mixed methods design,^23^ using semi-structured qualitative interviews to explore the views and experiences of individuals’ choice of, and satisfaction with, their risk-management strategy. Themes raised in these interviews informed the development of a validated questionnaire to quantify their importance. A mixed methods strategy was chosen to allow the benefits of the different components and to avoid the weaknesses of each from impacting on the overall findings. Data from both strands have been integrated throughout the analysis to provide quantification and explanation of each finding throughout and identify corroboration of results, or lack thereof, between different methodologies.

### Regulatory approvals

Research ethics approval was obtained from the UK National Research Ethics Service (ref 09/H1308/121) and local research governance approval was obtained.

#### Semi-structured qualitative interviews

Participants were identified and recruited from a locally held database or recruited prospectively from breast clinics between 2010 and 2012.

#### Eligibility

• Women judged to be at high breast cancer risk (≥30% lifetime risk) or a known carrier of a pathogenic mutation in *BRCA1* or *2*, attending family history clinic at a single UK teaching hospital.
• Offered a choice of RRS or surveillance.
• Partners of eligible women.

#### Recruitment

Purposive sampling was used, aiming to ensure recruitment of a range of ages of both women who had chosen RRS with and without reconstruction, and those who opted for enhanced surveillance.

Women were approached informally in the family history clinic to participate over a two-year period. Women expressing an interest were sent a formal study pack by post (comprising a letter of invitation, study information form, interview guide, consent form, study reply form and a freepost reply envelope).

#### Interviews

The interview schedule was based on existing literature from a systematic literature review (results not reported here) and expert opinion, focussing on the drivers for, and satisfaction with, choice of risk management.^10,24–40^ Women with partners were asked for permission to invite their partners to interview (by letter). Those whose partners responded positively were invited to participate by sending them a study pack (as above). Informed written consent was obtained from all participants. Interviews were digitally recorded and transcribed verbatim. Analysis was performed by two researchers (EM/SE) using the National Centre for Social Research “Framework” approach to identify themes in the data using Nvivo10 software.^41,42^ Recruitment ceased once data saturation had occurred.

### Questionnaire

#### Questionnaire design

A postal questionnaire was developed based on the relevant literature and themes identified in the interviews, in conjunction with an expert reference group and a patient focus group composed of four women who had faced this decision, to maximise face and content validity, usability and acceptability. A revised version was then piloted with two service users and modified according to feedback before use (Supplemental material).

#### Questionnaire recruitment

A two-stage invitation process was used to minimise the risk of psychological distress, as advised by the focus group participants, whereby an initial invitation letter to take part was sent out, followed by the questionnaire itself to positive responders. It was felt that the questionnaire might cause distress (as it covered some sensitive issues) to some women who might not wish to take part but might read the questionnaire if it was sent directly.

Participants were identified from a hospital database of women at high familial risk of breast cancer who had either been offered RRS or enhanced surveillance when attending family history clinic. For the purposes of determining the required number of respondents to the questionnaire survey, we assume the primary outcome for the survey was to estimate the proportion of responders who had high levels of decision satisfaction. Assuming a level of around 50% for this outcome, then, to estimate this proportion with a precision of ±15%, i.e. 95% confidence interval from 35% to 65%, would require 34 responders to the survey.

#### Questionnaire analysis

Questionnaires were sent out between 2012 and 2013. Descriptive statistics included medians and ranges for patient demographics. Where association between a demographic and a view were being assessed, Fisher’s exact test was used. Where comparison was drawn between, for example, women opting for surveillance versus surgery women, the Mann–Whiney *U* test was used. Data were analysed using IBM SPSS Statistics version 24. A *p*-value of <0.05 was regarded as being statistically significant.

## Results

### Qualitative interview demographics

A total of 32 women aged between 22 and 68 years (median age 44 years) were interviewed; 19 had undergone RRS, of whom 5 had a previous breast cancer diagnosis and 1 was awaiting RRS. Twelve had opted for enhanced surveillance. Of the 27 women who were in stable relationships at the time of their risk-management decision, 26 agreed to allow the study team to contact their partners for interview invitation. In total, seven partners (27%) responded positively and six were ultimately interviewed. Five were partners of women who had undergone RRS, two of whom had chosen not to have a reconstruction; the sixth was a partner of a woman who was awaiting RRS. The length of relationship at time of interview varied from 4 to 38 years, with risk-management decisions occurring after between 2 and 30 years together. Partner demographics were not collected but all were aged over 18 and all were male.

### Quantitative postal questionnaire survey demographics

Of 157 women invited to participate, 51 (32.5%) responded favourably and were sent the full questionnaire and, of these, 71% (36/51) (70.6%) returned the questionnaire; 17(47%) from women who had had RRS and 19 (53%) from women undergoing high risk surveillance. Demographics of respondents are shown in Table 1.

**Table 1:** Demographics of questionnaire respondents

	Screening (*n*=19)	Surgery (*n*=17)
Age	Median 40	Median 47
	Range 30–51	Range 29–69
Gene mutation	6 (31.6%) had gene mutations	15 (88.2%) had gene mutations
	• 1 *BRCA1*	• 5 *BRCA1*
	• 4 *BRCA2*	• 9 *BRCA2*
	• 1 unstated mutation	• 1 *ATM*
	13 had no proven mutation	2 had no proven mutation
Previous personal history of breast cancer	1 (5.3%) had a previous breast cancer diagnosis	6 (35.3%) had a previous breast cancer diagnosis
Partners	16 (84.2%) were in a relationship	16 (94.1%) were in a relationship
	1 (5.3%) was divorced	1 (5.9%) was divorced
	2 (10.5%) were single	
Children	14 (73.7%) had children	15 (88.2%) had children
	Children aged from 1–20 years	Children aged ranged from 2–48 years

### Synthesis of qualitative and quantitative findings

Thematic analysis of the interviews categorised data into four main themes, summarising their psychosocial outcomes and decision-making around risk management. These are summarised in Table 2 with representative quotes. The four themes are expanded below with data from the questionnaire to quantify findings and highlight variance between women choosing surveillance over RRS.

**Table 2:** Main interview themes with representative quotes

Theme	Participant quotes
**Perception of risk and the impact of increased risk**	
Women’s awareness and perception of risk	Perception of their own risk varied “*I was bound to have it already, I was going to die, basically history was going to repeat itself”* [ID2: BRCA, Surgery] “*Yes, my family’s got this predisposition thing, but that’s way off in the future and that’s how I feel, so you know, perhaps concerned enough to look out for signs but not to do something such as [surgery]”* [ID32: No demonstrated gene mutation, screening]
	Risk perception altered by family diagnosed with cancer: “*Both my mum and my auntie were both screened regularly, they both had mammograms done regularly… but it came in such an aggressive form with my mum and her sister that in between screenings they had missed it and it was too far advanced too quickly”* [ID20: No demonstrated gene mutation, initially screening, then surgery]
Women’s account of their risk being confirmed	Traumatised by the confirmation or discovery of their increased risk of breast cancer: “*…as if she’d told me I’d got cancer, that’s how bad I felt. I went to pieces, didn’t go to work for a week because I couldn’t sleep, I was panicking”* [ID2: BRCA, Surgery]
	Women with children felt guilty about the possible inherited risk they may have passed on: “*I felt as if I was handing them a poisoned chalice and I felt um, responsible and guilty although I know there’s no need, but that’s how I felt”* [ID28: BRCA, Screening]
	Gained some sense of control: “*Very keen obviously to get tested because I wanted to be in control of what happened not the other way”* [ID4: BRCA, Surgery]
	Difficult interfamilial relationships during the time that their risk was established. “*One of my sisters was angry with the way one of my other sisters had reacted to it and one of my sisters questioned my way of reacting to it”* [ID14: BRCA, surgery]
**Risk-management strategy decision making**	
Involving others in decision-making	Most women felt the decision had been their own. “*I tried to get feedback from my husband and a couple of close friends but each one of them wouldn’t commit on the decision, claiming it was entirely up to me*” [ID2: BRCA, surgery]
	Pressure from partners/family/doctors: “*I just went along with it and it all got to a point where I felt quite forced and quite pressurised into having surgery”* [ID6: BRCA, Surgery]
	Partner’s opinion of their involvement/role in decision-making: “*If she asked my opinion, I told her what I thought, but I only told her what I thought when she asked. It wasn’t my decision to make”* [Partner 1, surgery] “*I think I’d question erm, question her decision as to why she’d go down that route* [Partner 2, Screening] “*I might even suggest well, do you think it might be better if you if you had the mastectomy”* [Partner 5, surgery]
	Sense of uncertainty, even once decision had been reached. “*…like shall I shan’t I, yeah I’m going to do it, not I’m not and it’s still like that now”* [ID17: No demonstrated gene mutation, Screening]
Decision-making in women who chose surgery	Idea of risk being so high there was not really a choice: “*We’ve all got children, you know, what’s your choice really? For me, I didn’t really have a choice”* [ID3: BRCA, surgery]
	Felt that screening didn’t reduce the risk of cancer developing and concerns it would miss something: “*I didn’t want to wait for it to happen, I wanted to be proactive about it I guess”* [ID13: BRCA, surgery]
	Reconstruction was an important factor in making a decision for some.:“*I think I’d have felt totally different if I couldn’t have had the reconstruction*” [ID13: BRCA, Surgery]
Decision-making in women who chose enhanced surveillance	Idea that surgery was over-treatment: “*I might go through life and never get it and I might have this big operation you know for nothing”* [ID27: No demonstrated gene mutation, screening]
	Surgery could be reserved for when/if the situation changed: “*if I get breast cancer I’ll deal with it”* [ID12: BRCA, screening]
	Concern over the actual effects of surgery and the loss of their breasts. “*I wouldn’t put myself through that because I think, I just can’t imagine a woman without, er, bre…(breasts) I can’t imagine me without breasts”* [ID25: No demonstrated gene mutation, screening]
**Impact of risk-management strategy**	
Surgery	Trade-off between having breasts and having the constant worry of cancer: “*I wanted rid of them as soon as I’d made up my mind that they had to go because they just seemed… unnecessary”* [ID3: BRCA, Surgery]
	Relived cancer-related anxiety: *“breast cancer is not something I worry about more than any of my friends and yet in a sense people probably would expect me too but the fact that I’ve had the surgery really has took all that away”* [ID5: BRCA, Surgery]
	Importance of reconstruction: *“I think I’d have felt totally different if I couldn’t have had the reconstruction. I think I struggled enough as it was with the decrease in size”* [ID13: BRCA, surgery]
	Partners’ views of reconstruction: “*the reconstruction side of it was more for her than anything else”* [Partner 6: Surgery]: “*it is a bit weird they are there but they are not there”* [Partner 2, Surgery]
	Psychological impact of the operation: “*Cosmetically you look fine but dealing with the feelings and the sort of anguish that comes with reconstruction, it’s not, you never get back to normal”* [ID9: BRCA, surgery] “*I liked my back I think my back was probably (my) nicest bit and now I hate it”* [ID8: BRCA post Latissimus dorsi flap reconstruction] “*I am very worried about her, I suppose (more) psychologically than anything else, the impact it has had”* [Partner 6, surgery]
Surveillance	Confidence in the process: “*(MRI is) a more in depth test you know so that it shows up early”* [ID27: No demonstrated gene mutation, screening]
	Discomfort: “*it was very uncomfortable laid down on that, the bed was very hard, it were very difficult to keep still… it does make you sore for a few, a few days”* [ID45: No mutation, screening]
	Provides reassurance: “*I’m getting checks twice a year which is quite reassuring”* [ID30: No demonstrated gene mutation, screening]: “*they were, um, so quick and efficient and I got the results very quickly”* [ID51: No demonstrated gene mutation screening]
	Anxiety related to waiting for results: “*I get a bit apprehensive when I open the letter”* [ID45: No demonstrated gene mutation, screening]
**Support needs and partner relationship issues**	
Experience of support provided	Lack of face-to-face support from others who’d experienced it: “*I don’t think I was prepared for how I was going to feel afterwards [re surgery] and I think I would have liked to have spoke(n) to someone who had had it done”* [ID20: No demonstrated gene mutation, surgery] “*You don’t meet anyone else with the condition you don’t kind of get to talk it over with anyone else at all just, that’s it now we’ve told you, goodbye. I found that a bit weird I thought it was all a bit weird; the counselling was all one side”* [ID20: No demonstrated gene mutation, surgery]
	Variable experience of support received: “*on the care side we could not have asked for anything better… I don’t think I really have the right to say we have not had enough… as aware as we are that (name) needs the help we are also aware of how strained the services are”* [Partner 6, surgery] “*I don’t think there was any support at all thinking about it”* [Partner 4, surgery]
	Support for the partners: “*the only support I could have done with was my employers. Erm they gave me about three days, they did not understand the enormity of the surgery and the support that my partner needed”* [Partner 1, surgery]
Partner’s role in support provision	Both women and partners felt the partner’s role was that of support, despite their own feelings/opinions: “*He just wasn’t happy but he’ll just have to, you know, it’s my decision in the end, it’s my body, he said ‘but it’s your decision in the end but I don’t agree with it’”* [ID8: BRCA, surgery] “*Mostly I was there for support”* [Partner 1, surgery]
Impact on relationship	Variable impact on relationship with partner: “*I think he’s lost a bit of confidence in our relationship”* [ID2: BRCA, screening] “*probably brought us closer together to be honest and he really is supportive”* [ID32: No demonstrated gene abnormality, Screening]
	Impact of surgery on body image: “*She always turns round when she gets changed and things like that so it makes it, for me it makes it hard”* [Partner 3, surgery] “*he has very little interest in my breasts anymore*” [ID3: BRCA, Surgery]
	Physical symptoms: “*Sex is different. Don’t want it half of the time”* [ID1: BRCA, Surgery] “*sex is painful, is more painful than it used to be, I mean it never used to be so, it’s more painful so that makes me fearful of sex which obviously makes him nervous so that has affected it”* [ID3: BRCA, Surgery]
	Cause of stress for partner “*yeah, I think it worries him more than me actually. I mean, when I go for the MRI scan”* [ID70: No demonstrated gene abnormality, Screening]

#### Theme 1: perception of risk and the impact of increased risk

**Figure 1:**
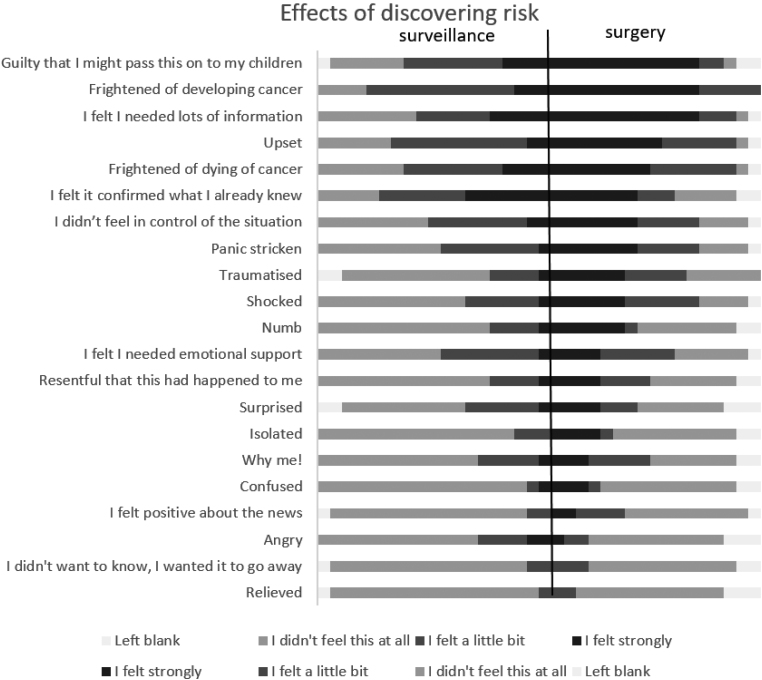
Christmas tree plot showing risk perception between the two risk-management groups in women at the time of being given their risk prediction or genetic diagnosis.

**Figure 2:**
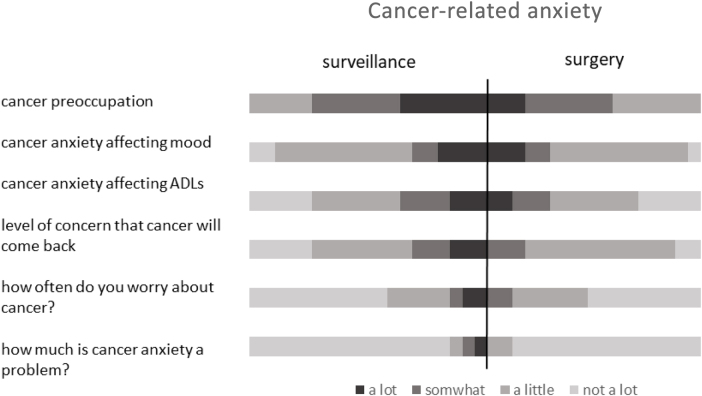
Current levels of cancer anxiety between risk-management strategy groups

Individuals’ perception of risk varied and was often based upon family members’ experience. Women undergoing surveillance estimated their risk of developing cancer as being lower than those who had undergone surgery (median 50% lifetime risk vs 80–90% for the RRM group) but this did not reach statistical significance (*p*=0.330). There was also a difference between the two groups in the level of impact that discovering their risk prediction or genetic diagnosis engendered (Figure 1), with a greater proportion of the surgery group recalling stronger feelings of fear and shock than those who chose surveillance (*p*=0.004). However, there was little difference in the level of cancer anxiety associated with their current risk-management choice (Figure 2).

The impact of RRM on risk perception was substantial for most women (Table 3) (*p*=0.001). It is apparent that some underestimated their risk before surgery and a few overestimated their residual risk after surgery.

**Table 3:** Perceived cancer risk pre and post RRM

Case	Perceived risk before RRM	Perceived risk after RRM	*p*-value
1	<1%	<1%	0.001
2	10%	10%	
3	10%	<1%	
4	50%	20%	
5	50%	20%	
6	80–90%	<1%	
7	80–90%	10%	
8	80–90%	<1%	
9	80–90%	10%	
10	80–90%	10%	
11	80–90%	50%	
12	80–90%	<1%	
13	80–90%	10%	
14	80–90%	10%	
15	80–90%	Left blank	
16	80–90%	10%	
17	80–90%	10%

RRM = risk-reducing mastectomy

**Figure 3:**
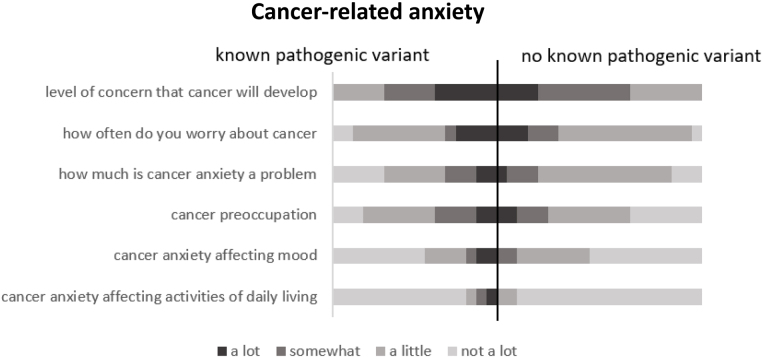
Christmas tree plot showing cancer-related anxiety in those with and without a known pathogenic variant

**Figure 4:**
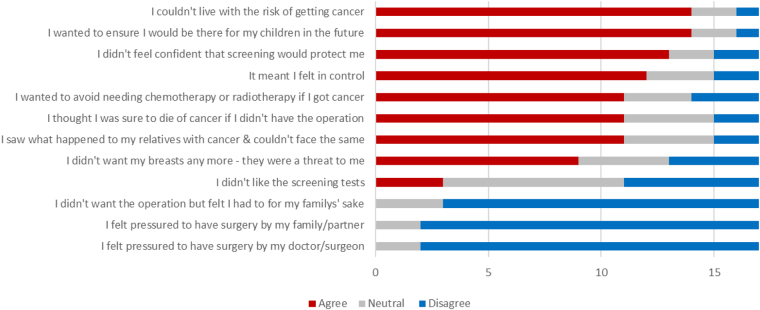
Reasons for choosing risk-reducing surgery

Women with a diagnosed gene mutation (*n*=20) had higher cancer-related anxiety than those without a known gene mutation (Figure 3). Those with a known pathogenic variant had a median risk perception of 80–90% (presurgery or surveillance) compared with a risk perception of 50% in those without, which was significantly less (*p*=0.027). They also described stronger feelings on confirmation of their risk (*p*<0.001) (Figure 4 supplemental).

Women with children frequently described feelings of guilt and 26/29 (89.6%) respondents with children admitted to feeling guilty about passing on the risk to their children.

#### Theme 2: risk-management strategy decision-making

For some women, the decision of how to manage their risk was straightforward, whereas others continued to feel uncertain after making their decision, particularly those without a recognised gene mutation. Overall, women broadly fell into one of three groups: those who chose RRS (“it’s the only thing that makes sense”), those who might consider it in the future (“not yet”) and those who felt they would never consider it (“no way!”).

A few of the women interviewed felt they had been pressurised into surgery/reconstruction, either by family or following the advice of their doctor. However, this was not substantiated in the questionnaire results, with no participants agreeing with either the statements “I felt pressurised to have surgery by my partner/family” or “I felt pressurised to have surgery by my doctor/surgeon”.

Many of the women who chose surgery felt they “had” to do it, either feeling the need to be there in future for their children or because they felt it was their only choice given the risk of cancer they had been presented with. In the questionnaire, 14/15 (93%) women with children stated positively that they had chosen surgery to be there for their children in the future. A greater number of women without children chose surveillance (14/19 had children in the surveillance group vs 15/17 in the surgery group), although numbers were small.

Women who opted for surgery more frequently noted that surveillance could not reduce the risk of cancer developing in the future and expressed concerns about the effectiveness of the test. This was also true in the questionnaire results, where 13/17 (76.5%) of women who chose surgery agreed with the statement “I didn’t feel confident that screening would protect me”. Women who chose surgery expressed greater concern about the possibility that surveillance could miss cancer compared with those who chose surveillance (66.67% vs 42.1%) or that interval cancers could develop (84.6% vs 47.1%), but neither result was significant. The surveillance group also appeared to be more optimistic about the likelihood that any cancer detected would be at an early stage than the surgical group, although this was not a significant difference (*p*=0.13).

For some women, the decision to undergo enhanced breast surveillance was an active choice and one with which they felt satisfied. For others, surveillance was merely accepted as an alternative to surgery (or to delay that decision).

Some felt that surgery was too drastic given that they may not ever develop cancer, with many stating that they would leave surgery until that situation changed. Others were more concerned with the actual effects of surgery and the loss of their breasts.

Given that cancer-related anxiety is a frequently quoted reason that women choose RRS, women were also asked to indicate how likely they thought they would be to survive cancer if it did develop. No significant difference was apparent in perception of cancer cure between women who chose surveillance and those who chose surgery (*p*=0.34).

Only 8 of 19 in the surveillance group recalled ever being offered RRS. This may reflect their lower level of risk such that RRS was not appropriate or their own lack of interest in the option such that it was not further explored by counsellors.

Of women who had chosen RRM, the reasons for the choice are presented in Figure 4. Figure 6 (supplemental) shows the attitudes of women who chose surveillance towards RRS, with the main drivers away from surgery being fear of the consequences of surgery and that it was more treatment than required, which fits with their generally lower risk perception.

#### Theme 3: impact of risk-management strategy

##### Surgery

In the interview group, 19 of the 20 (95.0%) women who opted for surgery had reconstruction, with 6 (30.0%) requiring planned or unplanned further surgery and 13 (65.0%) reporting postoperative complications. In the questionnaire group, 16/17 (94.1%) had reconstruction, 6 (35.3%) required planned or unplanned revision surgery and 10 (58.8%) reported postoperative complications.

The availability of reconstruction was an important factor in making a decision for some, but not all. There was a mixed reaction to the question, “I do not mind what my breasts look like as long as I got rid of the cancer risk” which was “not important” to some and “very important” to others. However 86.7% of questionnaire respondents felt it was very important to have a normal appearance when dressed.

From the partners’ perspective, reconstruction was described as being for the benefit of the woman and not from any desire of the partner for reconstruction.

Although most women expressed a welcome reduction in cancer worry following surgery, several felt that their cancer worry was merely reduced and not gone. Dealing with the loss of their natural breasts and the impact of the operation itself was a frequent cause of distress. Of the questionnaire respondents who had surgery, 13/17 (77.9%) rated the outcomes either okay, good or excellent; 22.1% rated results as poor or very poor. Sensation was poor in the majority (62.5%) and 24% rated their feel, comfort and appearance as either poor or very poor. Of the 17 patients who had reconstruction surgery, 8 had implant-only reconstruction, 5 had autologous-only and 4 had autologous latissimus dorsi reconstruction with implant; 9 (52.9%) of the patients who underwent reconstruction felt their reconstructed breasts were not their own.

**Figure 5:**
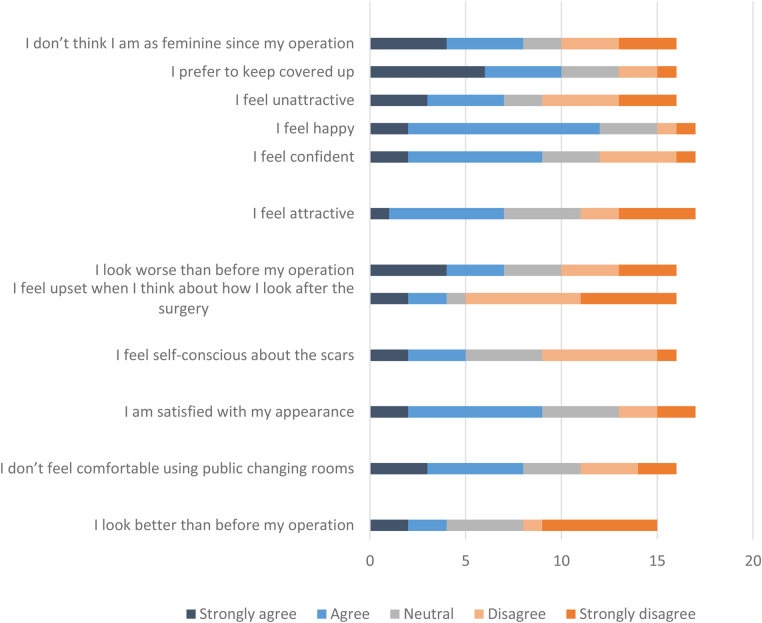
Women who chose surgery, body image scores after risk-reducing surgery

Some women felt guilty about having had RRS, agreeing that they did not feel as deserving as patients who had cancer. That being said, 100% of women who had RRS and who answered the question either agreed or strongly agreed with “I feel I made the right decision to have my breasts removed”; however, 3/15 (20%) would have chosen a different type of operation and 2 of 15 would have preferred, in hindsight, to have simple mastectomy without reconstruction. Figure 5 shows the questionnaire responses to questions about the outcomes of surgery, which shows the impact on body image, despite which the majority of women do not regret having RRS.

##### Surveillance

All interviewed women who chose surveillance over surgery felt they had made the right decision and were reassured by the surveillance process, with 17/19 of questionnaire respondents also agreeing with the statement “I feel confident that screening will identify any problems in my breast at an early stage” and less than half 9/19 (47.4%) were concerned about interval cancers or that surveillance would miss something (8/19; 42.1%). The vast majority felt MRI was a better test (than mammography), offering a more thorough assessment of their breasts and equating to greater levels of reassurance from the surveillance process.

Most women with experience of surveillance were circumspect about any inconvenience of the process, acknowledging the process as being necessary and worthwhile for the peace of mind it provided. The wait for results was described by some as a period of increased worry.

Of the questionnaire respondents, only 8/19 (42%) agreed with the statement “I felt anxious when attending for screening tests” and 11/19 (57%) agreed with the statement “I felt anxious when waiting for screening results”.

#### Theme 4: support needs and partner relationship issues

A number of women felt that peer-support would have been beneficial, both to aid decision-making and for ongoing support following their decision. Main sources of support differed between women in different RRS groups and between gene carriers and those without a demonstrated gene abnormality, with specialist healthcare professionals (clinic doctors and genetics nurses) playing an important role for more women who chose surgery or had a gene mutation.

Of the 17 questionnaire respondents who had surgery, 2 had contact with support groups, with 10 of the remaining 15 expressing a desire to have access to a support group. In the surveillance group, 1/19 had contact with a support group, with 6 of the remaining 18 wanting to have had access. Women who had undergone surgery preferred to have access to a support group upfront when they were making decisions about, and going through, the process of surgery. Women in a surveillance programme appeared to want support group access in the long term, likely reflecting their ongoing risk. Women with a gene mutation and those who chose surgery had a greater (unmet) demand for support groups, with a significant minority not wanting to engage in this facility.

Partners’ role in decision-making was seen as supportive by both the women and partners themselves, regardless of their own views. Those who opted for surgery described a greater impact on their relationships, with women who opted for surveillance tending to discuss the role of their partners less, perhaps reflecting a lesser need for support in this group. Very few partners seemed to have offered opinions or to have been involved in making risk-management decisions. This is reflected in the partner interviews and in the very low uptake by partners to take part in the study.

Of the questionnaire participants, 16 women were in a relationship at the time of surgery, of whom 8 felt it had changed since surgery. Five felt closer to their partner, none felt more distant. Five felt their physical relationship had changed in bad way, none felt it had improved. Three felt their relationship was better emotionally, two felt it was worse. No partnerships had broken down since surgery.

Half felt sexual activity had changed since surgery, with six feeling they had less interest in sex, two feeling they had more interest. Three felt their partner had less interest, one felt their partner had more interest.

Of those 13 women who chose surveillance who were in a relationship at the time they discovered their risk, 1 felt their relationship had changed (more distant and physically worse in a bad way) but 12 felt it had not. Of the 15 who were sexually active, none felt it had changed since finding out about their increased risk. None felt their partner’s attitude to sex had changed either.

## Discussion

### Risk

This study explored topics that have been previously reported, but in the context of enhanced surveillance with MRI, which was not in use at the time of previous similar studies.^25,26,31^

In line with published literature, discovery of risk was, for many, a traumatic event.^43^ Fear of cancer, treatment and death was frequently based on first-hand familial experience. For others it was confirmation of something they suspected and was accepted without shock. For some, establishing their risk enabled them to take action and regain control.

Discovery of risk and risk-management decision-making appear to be, for most women, events that they wish to move beyond, as described by both Lloyd and Lodder in their studies.^27,35^ For some women, RRS is an effective way to address their risk; for others, enrolling in a high-frequency surveillance programme provides this risk amelioration, allowing them, in the main, to accept their situation and move on with life. Choice of risk-management strategy needs to match women’s desire for metaphorical recovery and this will be determined by women individually, based on priorities, circumstances and perceptions that will usually be opaque to observers, be they family, friends or healthcare professionals.

Women with children frequently expressed feelings of guilt around passing on their risk. Another motivating factor driving some women to choose RRS is the idea that they can address the problem and move on more rapidly. That this process involves a mutilating procedure for which there are well-documented adverse outcomes is, for the majority of women who chose it, an acceptable cost for reducing the distress they experienced living at risk.

Women’s (recalled) reaction to being told they were at increased familial risk seemed to predict their subsequent risk-management decision. Women who reacted more strongly (for example, strong feelings of fear, panic and shock) were more likely to choose RRS than surveillance. High levels of distress upon discovery of risk did not correlate to high cancer-related anxiety and, whereas some studies found that cancer-related anxiety was more common in women who chose surgery, others, including this study, found it to be more common in women who chose surveillance.^35,44^ To our knowledge, this strength of feeling correlating with decision management has not been reported before.

### Impact of gene mutation

In keeping with published studies,^46^ those with gene mutations were more likely to choose surgery, reflecting the uncertainty of risk in those without a known mutation. While gene testing has improved significantly over the past few years, it is not yet possible to assume that women without a confirmed mutation are at lower risk.

### Factors impacting on decision making

This study supports the findings of previous research that cancer worry was the dominant force among women considering RRS.^16^ Some women with children felt they had no choice but to have surgery to reduce the risk of their children seeing them suffer with cancer, or worse, dying from it. The desire for control also motivated some women to choose surgery.

Women’s views of surveillance and surgery were very varied. Despite high numbers of recalls in the surveillance group (21%) compared with national published recall rates (8–17%),^6,7,46–48^ all women who had chosen surveillance felt it had been the correct decision. Views expressed by the surveillance group suggest that surveillance is not “a bridge to surgery” for many, but an active choice. It may also be true that healthcare professionals may have recommended surveillance over surgery for this group.

The majority of women who chose surgery felt that surveillance would not protect them. Most wanted to look normal when dressed, some wanted to look normal undressed too. Managing expectations, particularly pertinent to immediate breast reconstruction, is an important part of the risk-management decision-making counselling process. Without adequate information, women risk feeling disappointed by their choice.

### Outcomes of RRS and surveillance

Women, following surgery, described a vast array of feelings. Elation was common in the early postoperative period, as was a feeling of loss and a reluctance to look at the chest which, for a minority, persisted for years. Later on, feelings were equally mixed. Some still harboured significant cancer anxiety whereas others felt they had done everything they could and were no longer worried.

Reconstruction had mixed long-term outcomes. Some were delighted and described their reconstructed breasts as being an improvement over the originals. Others felt their breasts were not really their own. There were some who reported negative views of appearance, confidence and femininity following surgery, but they were in a small minority and of proportions broadly similar to other published studies.^18,36,49^ Franzoi demonstrated that body image is often reported as poorer with advancing age,^50^ but in this series there was no perceivable age-related difference.

Women were frequently quite matter-of-fact about the practicalities of their surveillance experiences and were happy with the care offered. Previous studies have highlighted the wait for results as a source of anxiety,^10,51^ but this was less apparent in this study. Some felt apprehension on receiving the results, but there was not the same distress between scan and results that had been apparent in these other studies. This may reflect the tendency of women who would find this more of a problem choosing RRS instead of surveillance.

That all responders reported feeling their decision had been the right one for them is reassuring. Although satisfaction with the decision to have surgery was high, this was not mirrored in satisfaction with reconstruction, perhaps reflecting the fact that mastectomy is accompanied by reduction in cancer worry, with no such effect related to the reconstruction, which is viewed more simply on the merits of its cosmetic outcome. In line with previous research, the various short- and long-term effects of surgery and the psychological impact of increased familial risk varied.^10–13^

### Interviews with partners

The overall response rate of the partner’s interviews was disappointing and merits exploration; this should be a focus of future research. One of the few similar studies involved interviews with partners of women who had reconstruction following mastectomy (for cancer).^52^ In their study women were not involved in a parallel study and perhaps the single invitation to participate in research carried more weight (for women and/or partners). Another difference is that women had been treated for cancer and perhaps partners felt more engaged or felt that they had more to comment on in interview.

All partners felt that the decision on how to manage risk was one that needed to be made by the affected partner, essentially independently. They all appeared to have an opinion but were reluctant to share this in case they swayed their partner in her decision. Similarly consistent was the finding that partners did not appear to want their (affected) partner to choose to have reconstruction for their benefit.

### Impact on relationship

Relationship change was common in the RRS group but rare in the surveillance group. Changes were mixed; some felt closer, some more distant and some felt their physical relationship had suffered. These changes could reflect postoperative recovery times, which, particularly for those having breast reconstruction, are not quick, but are likely to also capture the longer-term sequelae, including changes in body image, confidence and femininity. There is the possibility that some of the participants in the RRS group also had risk-reducing oophorectomy, which may have had an impact on their physical relationship.

The impact of supporting their partner through the operation and the (in some cases) lengthy recovery was significant. Access to time off and the need to use holidays from work to provide care was a frequent source of difficulty. Changes to sexual relationship were attributed to postoperative pain, tiredness and reduction in confidence after RRS. Some felt emotionally closer to their partner, having tackled the familial risk ‘“as a team”, but others felt their partner had become more distant. Watching their partner deal with the psychological impact of undergoing RRS was difficult and upsetting.

Several of the partners described simply wanting their partner alive and well, with or without breasts, with surprising ambivalence about breast reconstruction. The views of, and support provided by, partners strongly correlate with good psychosocial outcomes in published studies.^24,28^ Assessing partners’ views as part of the risk-management counselling process could be beneficial in providing extra support for women either without a partner or without a supportive, loving partner.

### Limitations

Limitations of this study include the self-selected nature of the women and their partners who chose to take part, introducing potential selection bias. In addition, despite purposive sampling, the younger and older age ranges were not well represented, nor were patients who opted for surgery without reconstruction. Partner interviews were small in number and all were partners of women who had chosen RRS, so may not be representative of the group as a whole. The main challenge in this study was recruitment, which may be due to the emotive nature of the subject.

The nature of semi-structured interviews means that the interviewer may introduce bias, although awareness of this phenomenon and the combination of two interviewers aimed to reduce this.

Previously, nonvalidated questionnaire tools have been shown to overestimate satisfaction.^53^ While significant time (from both researcher and focus group participants) and expertise have been spent validating this tool, it is possible that interpretation will improve with greater use. The wording of the questions may have also introduced error, although both the expert reference group and focus group were involved in selecting terminology for questions.

The questionnaire response rate was disappointing and meant that subgroup analyses were not possible, and that statistical analyses were likely to be underpowered. The focus group felt that participants needed to consent to being sent a questionnaire with potentially emotive and highly personal questions. This two-stage recruitment strategy is likely to have reduced the response rate considerably. In addition to the low response rate, there is the possibility of sampling bias and participation bias, which may limit the generalisability of our results to the target population.

Data were collected retrospectively, with variation in time from risk-management strategy decision-making to the interview, introducing recall bias, and some questions were hard to interpret without a baseline “norm” to act as a comparator. Comparing groups, be it surgery versus surveillance, or gene positive versus no gene mutation adds potential error. The individuals studied are a heterogeneous population, including those with and without known pathogenic variants, differing surgical procedures in the RRS group, and some of the surveillance group were offered (but declined) RRS, all of which may acts as confounders, limiting any firm conclusions.

A question was missed from the surveillance questionnaire that should, with hindsight, have been included, exploring the impact of recall. This would have provided a greater understanding of how, in challenging times, surveillance women balance the ongoing risk of cancer against the risks and benefits that are associated with RRS. Similarly, the questionnaire did not explore chemoprophylaxis. This, however, was not inadvertent. At the time of the questionnaire (2012) it was not routinely available, although it would be interesting in future work to look at women’s views of this modality of risk reduction and to see how it impacts on choices.

Finally, these data were collected from a single centre in 2011–2012, which may limit the generalisability of the results, and there have been advancements in surgical procedures and chemoprophylaxis in this time, although the authors feel these data still have relevance to modern practice.

## Conclusions

This study adds to the existing literature by measuring psychosocial outcomes at a time when options for risk management have recently changed. MRI surveillance is now widely available and represents a valid and effective risk-management strategy that, for some women, better matches their desire for risk amelioration and their tolerance of adverse effects of other options. This study also adds the views of partners, which have rarely been explored and particularly not since the addition of MRI surveillance.

These data demonstrate the need for high-quality information that is, ideally, tailored to the individual. Women deciding on surgery need to know the likely outcome of their individual choice and be provided with realistic expectations, in order that they can make a truly informed decision with which they remain satisfied in the long term.

Healthcare professionals involved in assessing risk, informing women of their risk-management options and guiding women through the actual process should explore all options available locally to facilitate a fully informed decision and well-supported journey.

Further work on a national scale may improve the generalisability and provide support to these data, on what is a very important but underresearched area of study.

We confirm that all authors have made a significant contribution at the following stages of the study: (1) the conception and design of the study, or acquisition of data, or analysis and interpretation of data, (2) drafting the article or revising it critically for important intellectual content and (3) final approval of the version to be submitted.

## Funding

This study was funded by the University of Sheffield.
